# Supplementary material for: Monogamy relations for relativistically causal correlations
Source: Nat Commun. 2025 Jan 2;16:269. doi: 10.1038/s41467-024-54855-1 (PMC11696493; doi:10.1038/s41467-024-54855-1)
Supplement: Supplementary file 1 — Supplementary Information [file 41467_2024_54855_MOESM1_ESM.pdf]

Supplementary Information: Monogamy relations for relativistically causal correlations

Mirjam Weilenmann[1]
[1] *Département de Physique Appliquée, Université de Genève, Genève, Switzerland*
(Dated: 5th December, 2024)

Supplementary Note 1. RELATIVISTIC CAUSALITY AND THE DEPENDENCY ON THE NUMBER OF SPATIAL DIMENSIONS

This section provides more details on the definition of relativistically causal correlations. In Section Supplementary Note 1 A, we give a formal definition of the multipartite non-signalling constraints, which allows for comparison with relativistic causality. In Section Supplementary Note 1 B, we contrast this with relativistically causal constraints, where we illustrate how the valid constraints in a specific setup may depend on the number of spatial dimensions and the arrangement of the involved parties.

A. Multipartite non-signalling conditions

In a setting involving n spacelike separated parties $1, \ldots n$, who can choose inputs X_i and obtain outcomes A_i, the *n-partite non-signalling constraints* can be written as [1]

$$\begin{aligned}\sum_{a_k} & P(\{a_1, \ldots, a_n\}|\{x_1, \ldots, x_n\}) \\ & = P(\{a_1, \ldots, a_n\} \setminus a_k | \{x_1, \ldots, x_n\} \setminus x_k)\end{aligned} \tag{S1}$$

$\forall\ k, \{a_1, \ldots, a_n\} \setminus a_k, x_1, \ldots, x_n$. For many arrangements of the parties, these constraints differ from the relativistic causality constraints of Definition II.1 from the main text.

B. Relativistically causal constraints in different arrangements and dimensions

To illustrate the role of Definition II.1 of the main text, we illustrate here that there are constellations of events, where it leads to constraints that are weaker than the ones that one would obtain from (S1). To see this for a specific arrangement, it is important to take *all* spatial dimensions into account when identifying whether a variable can potentially affect the correlations of others. We illustrate the dimension dependency by means of the example from Figure 1 from the main text in the following.

Example 1: *Let us first remark that if we consider the setup from Figure 1 from the main text in a world with just one spatial dimension, then spacelike separation of the three parties is sufficient for the three parties to display correlations that differ from* (S1).

In more spatial dimensions this is not the case anymore and in the following we show how the positions of (x, a), (y, b) *and* (z, c) *in Figure 1 affect this in different dimensions.*

Let us consider the example from Figure 1 of the main text but now take one additional spatial dimensions into account. For convenience of notation we choose units such that the speed of light is 1. Thus (in our reference frame from before) we have three events at coordinates $(t, u, v) = (0, 0, 0)$ *where* a *was generated from* x*, at* $(t, u, v) = (0, \frac{r}{2}, 0)$ *where* b *was generated from* y *and at* $(t, u, v) = (0, r, 0)$*, where* c *was generated from* z*. Note that* $r > 0$.

Now let us consider the boundary of the intersection of the future light cones of a *and* c*. This is the intersection of two cones parametrised by* r_a, s_a *and* r_c, s_c *as*

$$\mathcal{K}_a = \left\{ p_a \mid p_a = (t_a, r_a, s_a)\,,\ \sqrt{t_a^2 - r_a^2} \geq |s_a|,\ t_a \geq |r_a| \right\},$$
$$\mathcal{K}_c = \left\{ p_c \mid p_c = (0, r, 0) + (t_c, r_c, s_c)\,,\ \sqrt{t_c^2 - r_c^2} \geq |s_c|,\ t_c \geq |r_c| \right\},$$

namely $\mathcal{S}_{ac} = \mathcal{K}_a \cap \mathcal{K}_c$. *To characterise* $\mathcal{S}_{ac}$, *we first find the points that are simultaneously on the boundary of* $\mathcal{K}_a$ *and* $\mathcal{K}_c$, *namely*

$$\tilde{\mathcal{S}}_{ac} = \left\{ p_{ac} \mid p_{ac} = \left(t_{ac}, \frac{r}{2}, \pm\sqrt{t_{ac}^2 - (\frac{r}{2})^2} \right),\ t_{ac} \geq \frac{r}{2} \right\}.$$

Now $\mathcal{S}_{ac}$ *is the union of all light cones starting at points in* $\tilde{\mathcal{S}}_{ac}$. *Notice in particular that this set itself is not a light cone, i.e., its points are not within the future of a single point (as would be the case in* $1+1$*-dimensions). Namely, take* $\delta > 0$ *and compare the two points* $(\frac{r}{2}, \frac{r}{2}, 0)$, $(\frac{r}{2} + \delta, \frac{r}{2}, \sqrt{(\frac{r}{2}+\delta)^2 - (\frac{r}{2})^2}) \in \tilde{\mathcal{S}}_{ac}$. *Now the second point cannot be reached by* $(\frac{r}{2}, \frac{r}{2}, 0) + (\delta, 0, s)$, *since in a future light cone* $s \leq \delta$ *but* $\delta < \sqrt{\delta^2 + r\delta}$ *for* $\delta > 0$.

Now let us consider the light cone

$$\mathcal{K}_b = \left\{ p_b \mid p_b = \left(0, \frac{r}{2}, 0\right) + (t_b, r_b, s_b),\ \sqrt{t_b^2 - r_b^2} \geq |s_b|,\ t_b \geq |r_b| \right\}.$$

We observe that $\tilde{\mathcal{S}}_{ac} \subset \mathcal{K}_b$, *which is directly seen as* $r_b = 0$ *is allowed in* $\mathcal{K}_b$ *and thus also* $\mathcal{S}_{ac} \subset \mathcal{K}_b$. *Notice that since* $\mathcal{S}_{ac}$ *is the union of light cones that are all within* $\mathcal{K}_b$, *this will hold in all inertial reference frames. However, in contrast to the one dimensional case above, where* b *could be generated at a point* $(\frac{r}{2}, \frac{r}{2}, 0)$, *we cannot allow* b *to be generated at a point* $(r_0, \frac{r}{2}, 0)$ *with* $r_0 > 0$ *in the case of two spatial dimensions, since for* $\mathcal{S}_{ac} \subset \mathcal{K}_b$ *we need that* $r_0 \leq t_{ac} - \sqrt{t_{ac}^2 - \left(\frac{r}{2}\right)^2}$ *for all* $t_{ac} \geq \frac{r}{2}$. *A similar argument holds for* $r_0 < 0$.

The requirements on the setup in more than one spatial dimension are not only more rigid when considering the time dimension but also when trying to vary the spatial dimensions. Specifically, consider instead a starting position for b *that is slightly out of plane, i.e., for some* $\epsilon > 0$,

$$\mathcal{K}_b' = \left\{ p_b \mid p_b = \left(0, \frac{r}{2}, -\epsilon\right) + (t_b, r_b, s_b),\ \sqrt{t_b^2 - r_b^2} \geq |s_b|,\ t_b \geq |r_b| \right\}.$$

This implies that $\epsilon \leq t_{ac} - \sqrt{t_{ac}^2 - (\frac{r}{2})^2}\ \forall\ t_{ac} \geq \frac{r}{2}$. *Thus as* $t_{ac} \to \infty$, *we observe that* $\epsilon \to 0$. *In the line connecting* a *and* c, *the point* b *may however move, i.e., we can have*

$$\mathcal{K}_b'' = \left\{ p_b \mid p_b = \left(0, \frac{r}{2} - \epsilon, 0\right) + (t_b, r_b, s_b),\ \sqrt{t_b^2 - r_b^2} \geq |s_b|,\ t_b \geq |r_b| \right\},$$

as long as $-\frac{r}{2} \leq \epsilon \leq \frac{r}{2}$.

A further generalisation to three spatial dimensions proceeds along the same lines. The intersection of two future light cones in three spatial dimensions is a collection of future light cones: The region where two light cones,

$$\mathcal{K}_a^3 = \left\{ p_a \mid p_a = (t_a, r_a, s_a, q_a),\ \sqrt{t_a^2 - r_a^2 - s_a^2} \geq |q_a|,\ \sqrt{t_a^2 - r_a^2} \geq |s_a|,\ t_a \geq |r_a| \right\},$$
$$\mathcal{K}_c^3 = \left\{ p_c \mid p_c = (0, r, 0, 0) + (t_c, r_c, s_c, q_c),\ \sqrt{t_c^2 - r_c^2 - s_c^2} \geq |q_c|,\ \sqrt{t_c^2 - r_c^2} \geq |s_c|,\ t_c \geq |r_c| \right\},$$

intersect is in this case given by the future of any points within

$$\tilde{\mathcal{S}}_{ac}^3 = \left\{ p_{ac} | p_{ac} = \left(t_{ac}, \frac{r}{2}, s_{ac}, q_{ac} \right),\ t_{ac}^2 - (\frac{r}{2})^2 - s_{ac}^2 - q_{ac}^2 \geq 0 \right\}.$$

In general, the future of a set of variables $\mathcal{A}_{\mathcal{J}}$ is always a union of future light cones of some set of points $\tilde{\mathcal{S}}_{\mathcal{J}}$, that is a subset of a light cone $\mathcal{K}_{\mathcal{J}}$ that in some situations does not contain the full future light cones of all variables in $\mathcal{A}_{\mathcal{J}}$. This generalisation proceeds inductively in the sense that the intersection of a collection of light cones $\cap_i A_i$ is still a collection of light cones. Namely, $A_1 \cap A_2$ is a collection of light cones by the above, furthermore $A_1 \cap A_2 \cap A_3$ is obtained by intersecting each of the light cones in the collection with that of A_3 and then taking the union of the resulting light cones and so forth.

Supplementary Note 2. EXTREMAL CORRELATIONS AND MONOGAMY RELATIONS

In this section we provide more details on the extremal correlations for the scenarios considered in Figures 2 and 3 of the main text and provide further examples exhibiting monogamy relations.

A. Correlations in triangular setup with $Z = A \oplus B$

Notice that all relations imposed by relativistic causality are linear on the conditional distribution (no matter which light cone arrangement we consider). Additional conditions on the correlations like $P(ab|z) = 0$ if $Z \neq A \oplus B$, which can also be imposed as linear equalities for P, can thus be added and the respective polytope can be described in terms of its extremal vertices. In this case, this is made up from convex combinations of the two extremal points

$$P_1(abc|xyz) = \begin{cases} \frac{1}{2} & \text{if } z = a \oplus b,\ c = 0 \\ 0 & \text{otherwise.} \end{cases}$$

$$P_2(abc|xyz) = \begin{cases} \frac{1}{2} & \text{if } z = a \oplus b,\ c = 1 \\ 0 & \text{otherwise.} \end{cases}$$

Notice that due to the fine-tuned nature needed to enable $Z = A \oplus B$, imposing $P(ab|z) = 0$ if $Z \neq A \oplus B$ leads to the same polytope as imposing $P(ab|z) = \frac{1}{2}$ if $Z = A \oplus B$ in combination with the relativistic causality constraints.

Similar considerations can be made for other distributions and in particular when each of the six parties in the setup choose inputs and obtain outcomes. Let us consider, for instance, the example from [2] (Supplementary Note 5). There, a distribution $P_{\mathrm{HR}}(a_o y_o c_o | a_i y_i z_i)$ with binary inputs (outcomes) $A_i, Y_i, C_i (A_o, Y_o, C_o)$ is defined that is valid in the setting of Figure 1 of the main text. The distribution has marginals

$$P_{\mathrm{HR}}(a_o c_o | a_i y_i c_i) = \begin{cases} P_{\mathrm{perf}}(a_o c_o | a_i c_i) & y_i = 0 \\ P_{\mathrm{PR}}(a_o c_o | a_i c_i) & y_i = 1 \end{cases}$$

and $y_o = 0$ independently of all other variables, where $P_{\mathrm{perf}}(a_o c_o|a_i c_i)$ is $\frac{1}{2}$ iff $a_o \oplus c_o = 0$ and $P_{\mathrm{PR}}(a_o c_o|a_i c_i)$ is a PR-box, i.e., it is $\frac{1}{2}$ iff $a_o \oplus c_o = a_i \cdot c_i$. For A_o, C_o, Y_o and assuming uniformly random A_i, Y_i, C_i, these correlations are $P(a_o c_o|y_i) = \frac{1}{4} P_1(a_o c_o|y_i) + \frac{3}{4} P_2(a_o c_o|y_i)$ where $P_1(a_o c_o|y_i) = \frac{1}{2}$ iff $a_o \oplus c_o = y_i$ and $P_2(a_o c_o|y_i) = \frac{1}{2}$ iff $a_o \oplus c_o = 0$. Now we can ask whether such a distribution could also be implemented in the triangular setup of Figure 2 of the main text, with additional inputs for A_i, B_i, C_i and outcomes X_o, Y_o, Z_o. Thus, now interpreting A_o, C_o, Y_i as the respective variables in this setup, we can see that this type of influence from Y_i onto A_o, C_o is possible. A similar treatment as in the previous example,[1] namely, where we here require that for each x_i, z_i if $y_i = 0$, $P(01|y_i) = P(01|y_i) = 0$ and if $y_i = 1$, $3P(10|y_i) = P(00|y_i)$, $3P(01|y_i) = P(11|y_i)$ and $P(00|y_i) = P(11|y_i)$. Together with the relativistic causality constraints this forms a polytope of 65 extremal vertices that make up all distributions compatible with the triangle setup and with this marginal $P(a_o c_o|y_i)$. We can now check that there is no compatible distribution that has the same marginal for the variables $P(a_o b_o|z_i)$ or $P(b_o c_o|x_i)$, thus the same correlations cannot be implemented in the triangle (with additional inputs to A, B, C) along several sides at the same time. We do this by imposing these additional constraints and showing that there is no feasible solution to the resulting linear program. In order not to rely on linear programming, we further compute all possible marginals that we can observe when fixing $P(a_0 c_0|y_i)$ accordingly and find indeed that the same marginals are not possible for $P(a_o b_o|z_i)$ or $P(b_o c_o|x_i)$ under these constraints.

Another relevant distribution in the setting of Figure 1 of the main text is the one that was shown in [2] to violate monogamy of CHSH violation (see also main text for more on this). This distribution is defined as [2]

$$P(001|011) = P(110|011) = \frac{1}{2}, \quad P(011|110) = P(100|110) = \frac{1}{2}, \quad P(010|111) = P(101|111) = \frac{1}{2},$$
$$P(000|xyz) = P(111|xyz) = \frac{1}{2} \quad \forall (x_i, y_i, z_i) \notin \{(0,1,1), (1,1,0), (1,1,1)\},$$

all other probabilities are zero. For uniform X_i, Y_i, Z_i we can show that for this distribution $P(a_o c_o|y_i) = \frac{1}{2} P_1(a_o c_o|y_i) + \frac{1}{2} P_2(a_o c_o|y_i)$. Thus we impose that for each x_i, z_i if $y_i = 0$, $P(01|y_i) = P(01|y_i) = 0$ and if $y_i = 1$, $P(10|y_i) = P(00|y_i) = P(01|y_i) = P(11|y_i)$. In this case we obtain a polytope with 23 extremal vertices compatible with relativistic causality and these constraints. We further find again that the same marginal is not possible for

[1] Notice that $P(a_o c_o|x_i y_i z_i) = P(a_o c_o|y_i)$, where X_i, Z_i are the variables in the triangular setup.

$P(a_o b_o|z_i)$ or $P(b_o c_o|x_i)$ under these constraints. We check this again with a linear feasibility problem as well as by computing all possible marginals we can have for $P(a_o b_o|z_i)$ or $P(b_o c_o|x_i)$ in this case.

This means that these correlations can only be realised among three parties in the triangle setup of Figure 2 of the main text. At the same time having such a relativistically causal influence prevents the other parties from sharing such a distribution and in fact any distribution with such relativistically causal influence.

B. Full analysis of the compass setup

In this section we give a full description of the scenario displayed in Figure 3(a) of the main text. The relativistic causality constraints for this scenario are

$$\begin{aligned} P(ab|xy) &= P(ab|x) \\ P(ac|xy) &= P(ac) \\ P(bc|xy) &= P(bc|y) \\ P(a|xy) &= P(a) \\ P(b|xy) &= P(b) \\ P(c|xy) &= P(c) \end{aligned} \tag{S2}$$

$\forall\ a, b, c, x, y$. In this case we obtain only 82 extremal correlations (again obtained using PORTA [3] for the vertex enumeration). These come from 6 classes, the elements of which are equivalent up to exchange of A, X with C, Y and up to relabelling of inputs and outcomes. In the following we give an example from each class in functional form:

- Deterministic distributions (8 instances):

$$P(abc|xy) = \begin{cases} 1 & \text{if } a = 0, b = 0, c = 0 \\ 0 & \text{otherwise.} \end{cases}$$

- 3-party PR-box-like correlations (10 instances):

$$P(abc|xy) = \begin{cases} \frac{1}{4} & \text{if } a \oplus b \oplus c = x \cdot y \\ 0 & \text{otherwise.} \end{cases}$$

  This example is somewhat curious, since, while X doesn't affect the correlations between A, B and Y doesn't affect the correlations between B, C, X and Y *jointly* affect the correlations of A, B, C. This is indeed compatible with the space time setup in that the joint future of A, B, C is in the future of the joint future of X, Y.

- Monogamous correlations (8 instances):

$$P(abc|xy) = \begin{cases} \frac{1}{2} & \text{if } a \oplus b = x,\ c = 0 \\ 0 & \text{otherwise.} \end{cases}$$

- Type IV correlations (16 instances):

$$P(abc|xy) = \begin{cases} \frac{1}{4} & \text{if } a \oplus b = (y \oplus c) \cdot x,\ c = 0 \\ 0 & \text{otherwise.} \end{cases}$$

- Type V correlations (8 instances):

$$P(abc|xy) = \begin{cases} \frac{1}{4} & \text{if } x = a \oplus b, y = c \oplus b \quad \text{or} \quad x = a \oplus b, y = c \quad \text{or} \quad x = a, y = c \oplus b \\ 0 & \text{otherwise.} \end{cases}$$

- Type VI correlations (32 instances):

$$P(001|00) = P(010|00) = P(111|00) = 0,\ P(000|00) = P(100|00) = P(110|00) = \frac{1}{7},\ P(011|00) = P(101|00) = \frac{2}{7},$$
$$P(000|01) = P(111|01) = 0,\ P(001|01) = P(010|01) = P(011|01) = P(100|01) = P(110|01) = \frac{1}{7},\ P(101|01) = \frac{2}{7},$$
$$P(000|10) = P(011|10) = P(101|10) = P(110|10) = 0,\ P(010|10) = \frac{1}{7},\ P(001|10) = P(100|10) = P(111|10) = \frac{2}{7},$$
$$P(010|11) = P(100|11) = P(111|11) = 0,\ P(000|11) = P(001|11) = P(011|11) = \frac{1}{7},\ P(101|11) = P(110|11) = \frac{2}{7}.$$

[1] L. Masanes, A. Acin, and N. Gisin, Phys. Rev. A **73**, 012112 (2006).
[2] P. Horodecki and R. Ramanathan, Nature communications **10**, 1 (2019).
[3] T. Christof and A. Loebel, Porta-a polyhedron representation algorithm (1998).
